# Global ischemia induces stemness and dedifferentiation in human adult cardiomyocytes after cardiac arrest

**DOI:** 10.1038/s41598-024-65212-z

**Published:** 2024-06-20

**Authors:** Helen Jinton, Victoria Rotter Sopasakis, Linnéa Sjölin, Anders Oldfors, Anders Jeppsson, Jonatan Oras, Mathias Wernbom, Kristina Vukusic

**Affiliations:** 1 Department of Laboratory Medicine, Institute of Biomedicine, Sahlgrenska Academy, University of Gothenburg, Gothenburg, Sweden; 2 Department of Clinical Chemistry, Sahlgrenska University Hospital, Gothenburg, Sweden; 3 Department of Pathology, Sahlgrenska University Hospital, Gothenburg, Sweden; 4 Department of Cardiothoracic Surgery, Sahlgrenska University Hospital, Gothenburg, Sweden; 5 Department of Molecular and Clinical Medicine, Institute of Medicine, Sahlgrenska Academy, University of Gothenburg, Gothenburg, Sweden; 6 Department of Anaesthesiology and Intensive Care, Sahlgrenska University Hospital, Gothenburg, Sweden; 7 Department of Health and Rehabilitation, Institute of Neuroscience and Physiology, Sahlgrenska Academy, University of Gothenburg, Gothenburg, Sweden

**Keywords:** Heart, Regeneration, Cardiac arrest, Hypoxia, Atrioventricular junction, Dedifferentiation, Cardiomyocyte proliferation, Stem cells, Cell biology, Stem cells, Cardiology, Medical research

## Abstract

Global ischemia has been shown to induce cardiac regenerative response in animal models. One of the suggested mechanisms behind cardiac regeneration is dedifferentiation of cardiomyocytes. How human adult cardiomyocytes respond to global ischemia is not fully known. In this study, biopsies from the left ventricle (LV) and the atrioventricular junction (AVj), a potential stem cell niche, were collected from multi-organ donors with cardiac arrest (N = 15) or without cardiac arrest (N = 6). Using immunohistochemistry, we investigated the expression of biomarkers associated with stem cells during cardiomyogenesis; MDR1, SSEA4, NKX2.5, and WT1, proliferation markers PCNA and Ki67, and hypoxia responsive factor HIF1α. The myocyte nuclei marker PCM1 and cardiac Troponin T were also included. We found expression of cardiac stem cell markers in a subpopulation of LV cardiomyocytes in the cardiac arrest group. The same cells showed a low expression of Troponin T indicating remodeling of cardiomyocytes. No such expression was found in cardiomyocytes from the control group. Stem cell biomarker expression in AVj was more pronounced in the cardiac arrest group. Furthermore, co-expression of PCNA and Ki67 with PCM1 was only found in the cardiac arrest group in the AVj. Our results indicate that a subpopulation of human cardiomyocytes in the LV undergo partial dedifferentiation upon global ischemia and may be involved in the cardiac regenerative response together with immature cardiomyocytes in the AVj.

## Introduction

New cardiomyocytes are formed throughout life in humans^1,2^ but the mechanisms behind cardiac regeneration are still poorly understood. In most tissues, new cells are formed in stem cell niches. A potential cardiac stem cell niche was identified in the atrioventricular junction (AVj) of adult rat heart by using physical exercise for activation of stem cells in combination with DNA labeling with BrdU^3^. In human hearts, the AVj, at the insertion point of the mitral and tricuspid valves contain cells expressing cardiac embryonic stem cell markers and cardiomyocytes at different stages of development, and also display markers of migration and proliferation^4,5^. Cells expressing stem cell markers in the AVj may thus be of importance for the tissue homeostasis of the valvular apparatus as well as ventricles and atria. In line with this notion, impairment of this niche region was reported in patients suffering from end-stage heart failure^4^.

Another theory is that cardiac regenerative responses might be activated in surviving cardiomyocytes by global ischemia. Adult cardiomyocytes are terminally differentiated and do not divide. However, it has been shown in animal models of cardiac regeneration that some cardiomyocytes can re-enter the cell cycle^6,7^. In support of this theory, studies on animal models of cardiac injury, including myocardial infarction, suggest that regeneration is largely a result of proliferation of pre-existing cardiomyocytes^6,8^. Furthermore, it has been proposed that the remodeling in cardiomyocytes in response to pathological stimuli is intimately connected to cellular dedifferentiation, indicated by a loss of characteristics of mature (differentiated) cardiomyocytes and re-expression of genes from embryonic or fetal developmental stages^7^. According to this view, the co-existence of remodeling and dedifferentiation provides the necessary plasticity for survival, adaptation, and regeneration. Several studies on mammalian cardiomyocytes both in vivo and in vitro have provided evidence that dedifferentiation of cardiomyocytes may be a vital part of cardiac regeneration^6,9,10^. It is important to note that the term dedifferentiation in this context usually refers to a partial dedifferentiation process in the cardiomyocytes back to a more immature state, characterized by disassembly of sarcomeres and expression of embryonic and fetal genes prior to cell proliferation^7,9^. Interestingly, it has been shown that cardiomyocytes which have reverted to a more immature state after injury are characterized not only by cellular remodelling and a re-expression of early cardiac transcription factors but also by a re-expression of markers normally found in stem cells, which might contribute to the enhanced propensity of dedifferentiated cardiomyocytes to enter the cell cycle^7^.

Studies on apex resection in animals with a substantial regenerative capacity such as zebrafish and also newborn mice have provided strong evidence that existing cardiomyocytes are the dominant source for tissue repair in these models^8,10^. However, it is important to note that these results are not necessarily translatable to mammals, given the well-accepted lower potential for heart regeneration in adult mammals compared with zebrafish and amphibians^9,11^. Furthermore, the regenerative response of cardiomyocytes in human hearts to global ischemia is still not fully understood. Therefore, the aim of the present study was to map the cellular response to global ischemia in adult human hearts with reference to remodeling and regenerative processes.

This study is based on a unique material consisting of human cardiac muscle biopsies from multi-organ donors. The first group of biopsies was collected from donors with cardiac arrest that were successfully resuscitated but later declared brain dead due to ischemic cerebral edema. For comparison, a control group of hearts from organ donors without cardiac arrest, declared brain dead after primary brain injury, was used. This tissue collection provided a unique opportunity to study the regenerative mechanisms after a period of global ischemia in the adult human heart followed by approximately 2–4 day of reperfusion, before organ donation.

To investigate the cellular expression of stem cell-, hypoxia- and proliferation associated biomarkers, immunohistochemistry was used. The embryonic cardiac stem cell markers WT1, NKX2.5 and SSEA4^12–14^ were analysed along with the efflux protein MDR1, which is expressed by the side population of cardiac progenitors that has been identified in human cardiac tissue^15,16^. The hypoxia related marker Hif1α, which is expressed in cell nuclei under low oxygen conditions^17,18^ was included. To investigate the potential development of new cardiomyocytes, the proliferation markers Ki67 and PCNA^19–21^ were combined with the sarcomeric protein cardiac Troponin T (cTnT) and the cardiomyocyte specific nuclei marker PCM1^22^.

## Materials and methods

### Cardiac biopsies

Research ethics approval was granted by the Research Ethics Board at the Sahlgrenska Academy, University of Gothenburg, Sweden, and all study procedures followed the ethical guidelines of the declaration of Helsinki. A unique material consisting of human cardiac biopsies from explanted hearts from multiorgan donors were used in this study. The hearts were not transplanted but used for aortic homograft. Informed consent was obtained from the donors via organ donor register or next of kin. Table 1 summarizes the clinical background of each donor including age, sex, and the time of hypoxia period and from CPR to donation. Detailed Supplementary Table 1 summarizes e.g. cause of death, mechanisms behind the cardiac arrest, and results from the echocardiography for each donor. The biopsies were harvested from donors (age 19–75) who had suffered a prolonged cardiac arrest (N = 15) and a control group that consisted of donors without documented cardiac arrest (N = 6). Biopsies were obtained from two different locations in each heart; 1. Left atrioventricular junction (AVj) located at the lateral base of mitral valve, between left atrium and ventricle that represents the previously proposed cardiac stem cell niche^3–5^, and 2. transmural left ventricular myocardium (LV) tissue from the lateral middle region.

**Table 1:** Clinical background of included multi-organ donors.

Donor	Sex	Age	Cardiac arrest	Onset to donation	Onset of CPR to ROSC	Medical history
1	F	58	No	2 days	–	Ischemic heart disease, hypertension, atrial fibrillation
2	F	31	No	2 days	–	No known disease
3	M	62	No	3 days	–	Atrial fibrillation, MAZE surgery
4	M	74	No	2 days	–	No known disease
5	F	75	No	3 days	–	Hypertension
6	M	63	No	2 days	–	Hypertension
7	F	44	Yes	3 days	20 min	No known disease
8	F	63	Yes	2 days	30 min	IHD, hypertension, Diabetes type 2
9	F	19	Yes	2 days	20 min	Asthma
10	F	43	Yes	3 days	50 min	Hypertension
11	M	52	Yes	3 days	30 min	Hypertension, Diabetes type 2
12	M	63	Yes	3 days	5 min	Hypertension
13	M	54	Yes	3 days	10 min	No known disease
14	M	69	Yes	4 days	20 min	No known disease
15	M	19	Yes	4 days	40 min	No known disease
16	M	21	Yes	3 days	20 min	No known disease
17	M	46	Yes	3 days	30 min	No known disease
18	M	30	Yes	3 days	20 min	No known disease
19	M	24	Yes	3 days	30 min	No known disease, elite athlete
20	M	31	Yes	3 days	50 min	Obesity
21	F	53	Yes	3 days	75 min	No known disease

Summary of the clinical background of the organ donors included in the study.

*F* female, *M* male, *CPR* cardiopulmonary resuscitation, *ROSC* return of spontaneous circulation.

### Histology

Tissues were embedded in Tragacanth mounting medium (Histolab products AB, Gothenburg, Sweden), frozen in liquid nitrogen and stored at − 80 °C. Using a cryostat (Leica CM1950), biopsies were cut at − 20 °C into 7 μm sections and mounted on SuperFrost plus glass slides (Thermo Fisher Scientific). For histological analysis, tissue sections were stained with Hematoxylin and eosin.

### Immunohistochemistry (IHC)

Tissue sections were fixed in − 20 °C acetone for 10 min, and thereafter washed in phosphate buffer saline (PBS). The background was blocked using a solution consisting of 1% bovine serum albumin, 0.3 Triton X100 and 5% goat serum (Invitrogen, Carlsbad, CA, USA) for 30 min at room temperature (RT). This was followed by another wash in PBS, before the primary antibodies were added. In each protocol, three primary antibodies were used in different combinations according to Table 2. Primary antibodies were incubated overnight at 4 °C. Tissue sections were washed, and secondary antibodies added for one hour at RT; goat anti mouse Alexa Flour 546 or 647, goat anti rabbit Alexa Flour 546 or 647 (Invitrogen). The cTnT antibody was conjugated with Alexa 488 using a Zenon Kit (Invitrogen), to make it possible for the use of two mouse primary antibodies. All the samples were fixated using Histofix (Histolab, Gothenburg) for 15 min and washed. The mounting medium used was prolong gold antifade reagent with DAPI (Invitrogen). Isotype controls, corresponding to the primary antibodies, were used and did not show any specific staining.

**Table 2:** Antibodies used for immunohistochemistry.

Biomarker	Primary antibody	Concentration (µg/ml)	Dilution	Manufacturer	Catalogue number
WT1	Rabbit monoclonal IgG	0.2	1:250	Abcam	ab89901
SSEA4	Mouse monoclonal lgG3	0.5	1:60	eBioscience	14-8843-80
MDR1	Rabbit monoclonal IgG	0.3	1:250	Abcam	ab170904
cTnT	Mouse monoclonal IgG1	0.2	1:50	Thermo Fisher^a^	MS-295-P1
HIF1α	Mouse monoclonal IgG1	1.0	1:100	Novusbio	NB-100-131
Ki67	Mouse monoclonal IgG1	0.2	1:200	Santa Cruz^b^	sc-2390
PCM1	Rabbit polyclonal IgG	0.3	1:400	Atlas	HPA023374
PCNA	Mouse monoclonal IgG2a	1.0	1:1000	Abcam	ab-29-100
NKX2.5	Rabbit polyclonal IgG	1.0	1:300	Abcam	ab35842

Summary of the primary antibodies used in this study for immunohistochemistry.

^a^Thermo Fisher Scientific.

^b^Santa Cruz Biotechnology.

### Fluorescence microscopy and image analysis

To analyse the tissue sections, an Eclipse Ti inverted microscope (Nikon Corporation, Tokyo, Japan) was used. For histological analysis, images were photographed with a Nikon DS-2Mv camera, and fluorescence for IHC with an Andor Zyla camera. To cover the atrioventricular junction and the ventricular wall, images were captured by taking 7 × 7 photos of fields with a 20× objective. Four different channels were used (green Alexa 488, yellow Alexa 546, red Alexa 647 and blue DAPI). The expression of biomarkers was analysed by using the software Image J (v1.53, Fiji distribution), which was also used to stitch together the 7 × 7 photos and thus create a large composite photo. For each channel, the different ranges were set to eliminate most of the background. Representative images were cropped to show detailed staining of the different biomarkers.

### Quantification of PCM1 positive (PCM1+) twin nuclei and WT1+ nuclei

In the PCM1 stained sections, it became apparent that some cardiomyocytes contained two nuclei, here termed “twin nuclei”. For the purposes of this article, the definition of twin nuclei is two PCM1+ nuclei near each other with no visible cytosolic space between them. For control group; AVj N = 3, LV N = 4, and cardiac arrest AVj N = 13 and LV N = 13. The number of WT1+/cTnT+ cell nuclei were also counted, control group N = 4 and cardiac arrest N = 12.

For every donor, 1–4 tissue sections were stained and analysed. For each stained section, the number twin nuclei or WT1+/cTnT+ cells were counted in large images; 49 photos of fields with a 20× objective. The area of each large image was 5.8 mm^2^. A few sections were excluded due to artefacts. All the images were handled systematically, by using a grid function in the Image J software where the positive cells were counted manually. The cell numbers were processed in Microsoft Excel and a mean value for each location and group was calculated.

### Ethics approval

The study was approved by the Research Ethics Board at the Sahlgrenska Academy, University of Gothenburg, Sweden, following the Helsinki Declaration.

### Consent to participate

Documentation of informed consent from the multi organ donors, stating that their organs could be used for other medical purposes than organ donation.

## Results

### Histology of left atrioventricular junction (AVj) and left ventricle (LV)

**Figure 1:**
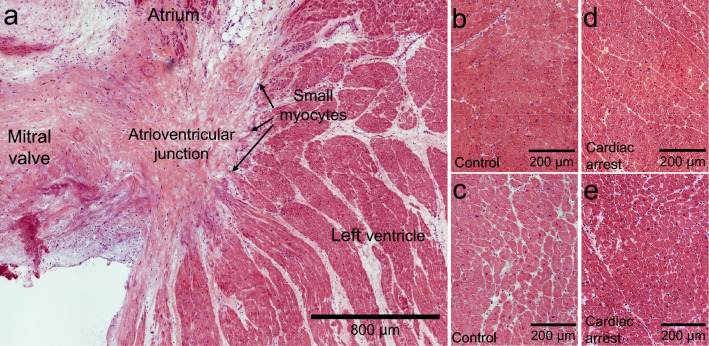
Histological overview of the human Atrioventricular junction (AVj) and left ventricle. Tissue sections were stained with Hematoxylin–Eosin. (**a**) AVj is located between the left ventricle and atrium near the mitral valve and consists of fibrotic tissue. Arrows point out the ventricular border, where islands of small cardiomyocytes reside. (**b**) and (**c**) showcase left ventricle tissue from two representative control donors. These biopsies were collected from the lateral side of mid-region of the muscular wall. (**d**) and (**e**) display the morphology of left ventricle tissue after cardiac arrest from two representative donors.

An overview of the AVj region is shown (Fig. 1a), where the fibrous anulus pulposus structure connects the mitral valve with the left atrium and ventricle. Most of the junction is comprised of extra cellular matrix rich tissue. Islands of small cardiomyocytes were dense at the border to myocardium. No histological differences were observed between the cardiac arrest- and control tissue when comparing LV myocardium (Fig. 1b–e) or the AVj (Suppl. Fig. 1).

### Expression of cardiac stem cell markers and hypoxia responsive factor Hif1α in the AVj

**Figure 2:**
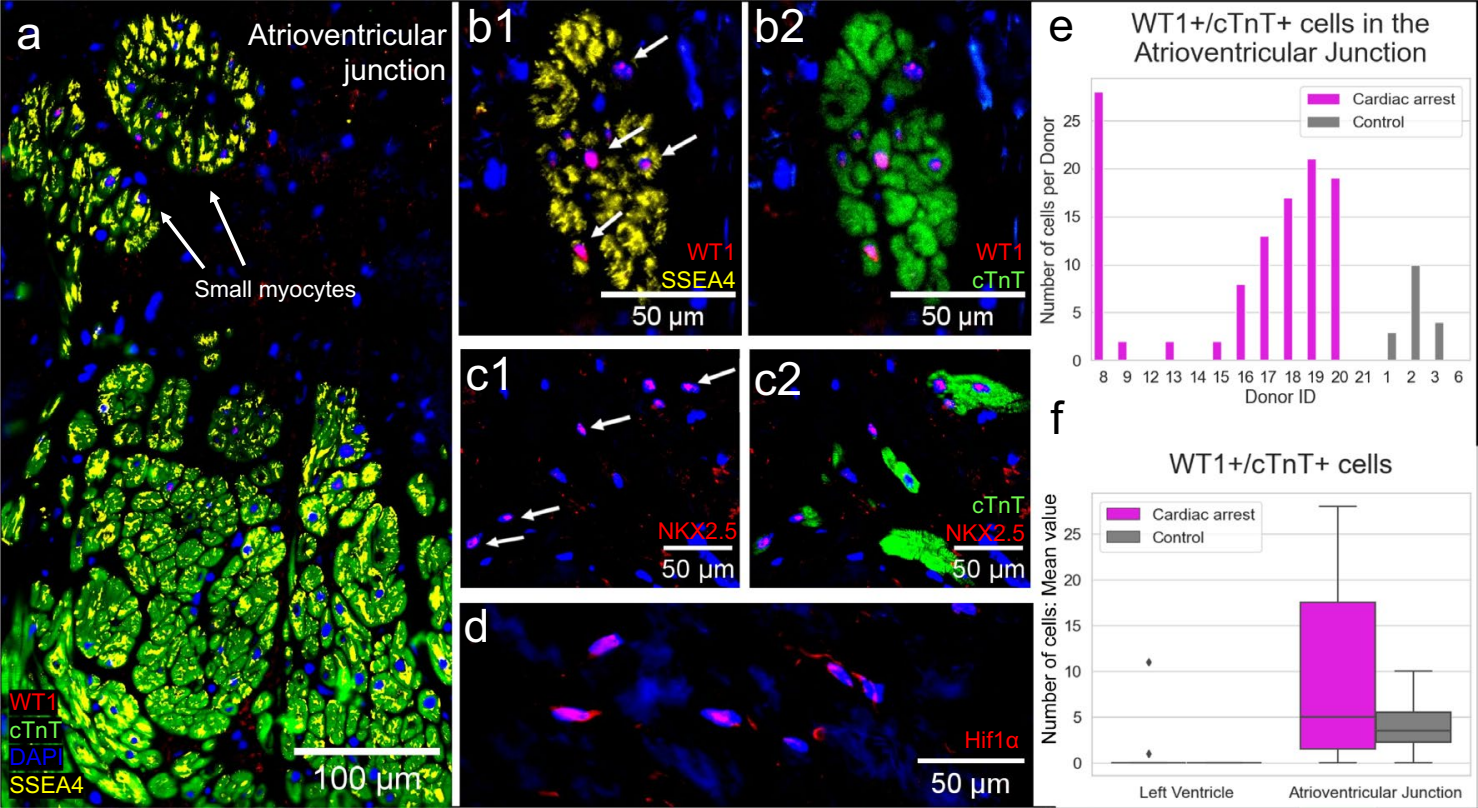
Immunohistochemistry showing expression of cardiac stem cell related markers SSEA4 (yellow), WT1 (red) and NKX2,5 (red) and hypoxia related marker Hif1α (red) in the atrioventricular junction (AVj) of cardiac arrest donors. Nuclei were stained blue with DAPI. (**a**) An overview of the AVj of a representative cardiac arrest donor, with the small cTnT+ cardiomyocytes that co-express SSEA4 and WT1. Larger cardiomyocytes further down into the myocardium express progressively less SSEA4 and WT1. (**b**1–**b**2) Close up of small cardiomyocytes in the AVj, expressing SSEA4 and WT1. (**c**1–**c**2) NKX2,5 expression in cells with and without cTnT expression. (**d**) Hif1α positive cell nuclei. Nuclei were stained blue with DAPI. The number of WT1+/cTnT+ cells were counted in 5.8 mm^2^ large images for each donor. (**e**) Number of WT1+/cTnT+ cells for each donor in AVj tissue, control group N = 4 and cardiac arrest N = 12. (**f**) Mean value number of WT1+/cTnT+ cells in both groups and locations.

In the proposed stem cell niche region AVj, expression of stem cell markers was detected in both groups of biopsies. Overall, the expression appeared to be more pronounced in the cardiac arrest group compared to the controls (Supplementary Fig. 2). The strongest expression of stem cell related marker SSEA4 was found among the small myocytes, in the AVj (Fig. 2). The expression decreased with the increased distance from the AVj. Cardiac stem cell related transcription factors WT1 and NKX2.5 were expressed in the islands of small myocytes at the myocardium border in AVj. Cells with WT1+/SSEA4+/cTnT+ signature as well as NKX2.5+/cTnT+ cells were detected suggesting cardiomyocyte specific progenitors (Fig. 2b1–c2). Nuclear expression of Hif1α was found in the anulus fibrosus of the AVj (Fig. 2d). No nuclear expression of the Hif1α could be detected in the atrial and ventricular parts of AVj or in the left ventricle, in either group (data not shown). WT1+/cTnT+ showed the highest numbers in the AVj of cardiac arrest group (Fig. 2e,f). In LV there were no WT1+/cTnT+ cells, except for few cells in tissue from 2 donors in cardiac arrest group.

### Reduced cardiac Troponin T expression and upregulation of cardiac stem cell markers in cardiomyocytes after cardiac arrest

**Figure 3:**
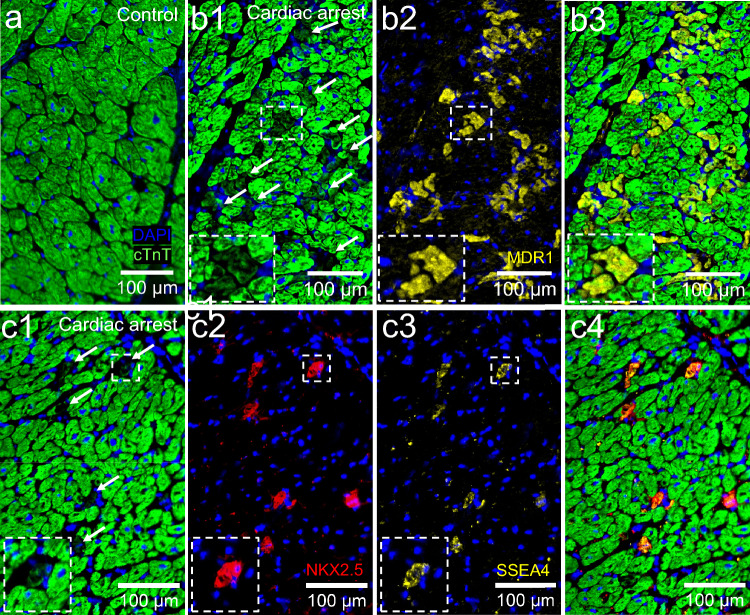
Immunohistochemistry of left ventricular (LV) tissue showing reduced expression of the sarcomeric protein cardiac Troponin T, cTnT (green) in single cardiomyocytes and upregulation of cardiac stem cell related markers; MDR1, NKX2,5 (red) and SSEA4 (yellow) in the cardiac arrest group. (**a**) LV from a representative control, with equal expression of cTnT in all cardiomyocytes. (**b**1–3) LV from a representative cardiac arrest donor with a subpopulation of cardiomyocytes (arrows) showing a decreased expression of cTnT and expression of MDR1. (**c**1–4) Reduced cTnT expression (arrows) in cardiomyocytes overlapped with the expression of NKX2.5 and SSEA4 in single cardiomyocytes in the cardiac arrest group. Nuclei were stained blue with DAPI.

The expression of cardiac Troponin T (cTnT) in the LV tissue was equally distributed in the cytoplasm of the cardiomyocytes. However, individual cardiomyocytes with a reduced expression of cTnT were identified in the cardiac arrest group (Fig. 3a,b1). This subpopulation of ventricular cardiomyocytes with low cTnT showed an expression of MDR1 (Fig. 3b2,b3). Furthermore, cardiomyocytes in the cardiac arrest group also expressed NKX2.5 (Fig. 3c1–c4). These NKX2.5+ cardiomyocytes also showed reduced cTnT expression as well as an expression of SSEA4. WT1 was not expressed by LV cardiomyocytes in either of the groups (data not shown). No cardiomyocytes with a decrease in cTnT expression could be found in the left ventricle of the control group, and there was no expression of MDR1, NKX2.5, or SSEA4 (data not shown). WT1 expression in LV was found in cTnT− cells, between cardiomyocytes, in the cardiac arrest group in tissue from donors; 8, 9, 14, 15 and 16 (data not shown).

### Expression of proliferation-related markers PCNA and Ki67 in the AVj

**Figure 4:**
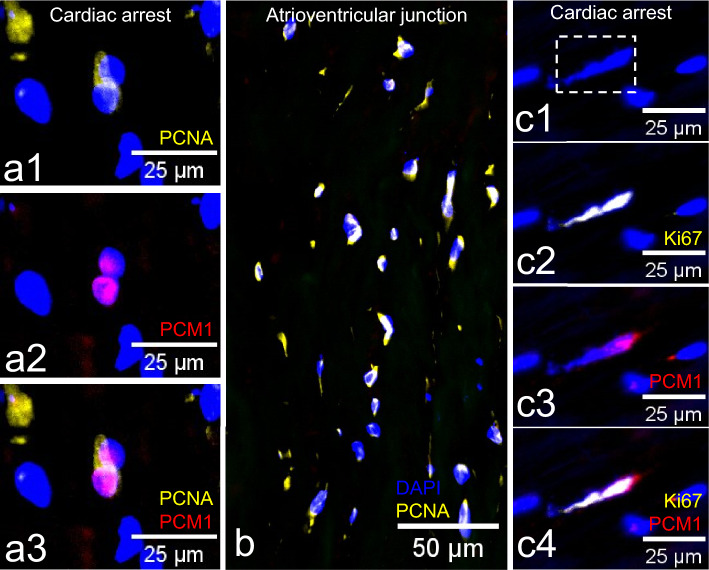
Expression of proliferation related markers PCNA and Ki67 (yellow), together with cardiomyocyte nuclei marker PCM1 (red) in cardiac arrest group. (**a**1–3) Cell nuclei with expression of both PCNA and PCM1 in the atrioventricular junction (AVj). (**b**) Many PCNA + nuclei in the AVj from a representative donor after cardiac arrest (**c**1–4) Cell nuclei in the AVj where one of the two Ki67+ nuclei co-expresses cardiomyocyte nuclei marker PCM1 indicating asymmetric cell division.

PCNA was expressed by many nuclei in the AVj in both groups (Fig. 4). However, co-expression of PCNA and PCM1 in cardiomyocyte nuclei was found only in the cardiac arrest group (Fig. 4a1–a3). Ki67 showed a similar pattern, where rare Ki67+/PCM1+ cells were found in the AVj after cardiac arrest (Fig. 4c1–c4). No expression of PCNA or Ki67 was detected in PCM1+ nuclei of the left ventricular tissue.

### Cardiomyocytes with PCM1+ twin nuclei

**Figure 5:**
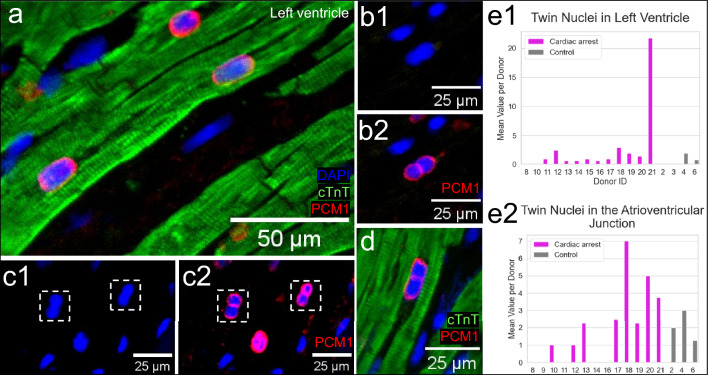
Detection and quantification of rare cardiomyocytes with PCM1+ twin nuclei (red) after cardiac arrest. (**a**) Overview of a binuclear cardiomyocyte with parted nuclei in left ventricle. (**b**,**c**) Cell nuclei with and without PCM1 expression from the ventricular myocardium in cardiac arrest group. PCM1 expression pattern showed a split between two nuclei, close together that was not observed with DAPI. (**d**) cTnT+/PCM1+ cardiomyocyte from the cardiac arrest group with twin nuclei. (**e**1–2) Bar graphs displaying the higher mean value of PCM1+ twin nuclei/donor, in cardiac arrest group compared to controls in both left ventricles and the atrioventricular junction. Twin nuclei were counted in 5.8 mm^2^ large images of 49 photos of fields with a 20 × objective/tissue section. 2–4 tissue sections were stained and counted per donor.

Nuclei in cardiomyocytes are normally separated (Fig. 5a). Representative PCM1+ twin nuclei are shown (Fig. 5b1,b2,c1,c2,d). The number of twin nuclei was higher in the cardiac arrest group in both locations, with high biological variation between the donors (Fig. 5e1–e2). In most cases, twin nuclei could not reliably be distinguished as two when stained only with DAPI but were revealed with by the PCM1 expression.

## Discussion

Animal models have shown that cardiomyocytes can indeed regenerate. However, animal studies are not always directly transferable to a human setting. Thus, it is essential to assess regenerative processes in human heart when possible. In this study, we evaluated the expression of several early cardiac stem cell- and proliferation- associated biomarkers in adult human cardiac tissue from the left ventricle (LV) and the potential stem cell niche region the atrioventricular junction (AVj)^3–5^. Specifically, we sought to investigate whether global ischemia, caused by cardiac arrest, activate regenerative processes such as cardiomyocyte remodeling and cell renewal in the adult human heart.

When damage occur in cardiac tissue, the result is often an increase in fibrosis, hypertrophy, adipose tissue infiltration, lipofuscin accumulation or nuclei fragmentation. We expected histological changes in the cardiac arrest group, but no clear difference was observed by the systematic analysis of the tissue. The time window, 2–4 days between the cardiac arrest and organ donation, seems too short for a histological remodeling of the normal myocardium to be demonstrated.

To further investigate potential differences between the two groups, the expression of cTnT, a part of the sarcomeres of cardiomyocytes, was studied. As expected, an even distribution was found in LV from the control group. In contrast, after cardiac arrest individual cardiomyocytes with a decreased expression of cTnT were discovered. Moreover, our results show that these cardiomyocytes with low cTnT upregulated the expression of the stem cell associated biomarkers. This is an interesting similarity to transient cardiomyocyte dedifferentiation, which is characterized by structural remodelling of the sarcomeres including decreased levels of cTnT^7,9^. We have previously reported that MDR1, SSEA4 and WT1 are expressed in small immature myocytes in the suggested hypoxic stem cell niche in the human AVj but not in LV from the same hearts ^4^. Upregulation of these early cardiac biomarkers in a subpopulation of LV cardiomyocytes post ischemia supports the idea of dedifferentiation in existing cardiomyocytes. Furthermore, a study using brief non-lethal myocardial ischemia–reperfusion model in sheep reported that MDR1 was upregulated both after 3 and 48 h following reperfusion^23^. The authors proposed MDR1 as an early biomarker whose activation plays a pivotal role for cell survival. The expression of MDR1 in human LV following reperfusion after cardiac arrest in our study is consistent with a prolonged expression in response to global ischemia.

In addition, we found expression of the early cardiac transcription factor NKX2.5 in the LV cardiomyocytes with reduced cTnT interpreted as a potential reprogramming of adult cardiomyocytes into a more immature phenotype. NKX2.5 expression was detected in dedifferentiating rat cardiomyocytes in culture^9^ and is known to lie upstream of many essential genes for heart development^13^. Furthermore, in zebrafish, it was shown that activation of NKX2.5 was required for not only adult myocardial repair but also to provoke the associated proteolytic pathways of sarcomere disassembly as well as the proliferative response for cardiomyocyte renewal^24^. In line with this background, we suggest that the increase in NKX2.5 in combination with a decrease in cTnT expression may signify a remodeling process. Collectively, a reduction in cTnT and upregulation of stem cell associated biomarkers after an episode of global ischemia followed by few days of oxygen supply indicate a remodeling of the LV cardiomyocytes.

Intriguingly, hypoxia has been shown to induce dedifferentiation of early committed cells into pluripotency^25^. The fact that no nuclear Hif1α expression was observed in the LV after cardiac arrest despite the increased MDR1 expression after 1–4 days of reperfusion is likely due to the very short half-life (~ 5–8 min) of Hif1α after return to normal oxygen levels^26,27^. Under normal conditions Hif1α is expressed in the cytoplasm^18^, but when the oxygen levels drop Hif1α is instead accumulated in the nuclei^17^. Beyond its function as a transcriptional regulator for the cellular response to hypoxia, Hif1α plays a role in the activation of genes related to tissue repair^28^. In contrast to the findings in LV, nuclear Hif1α expression was detected in non-cardiomyocytes in the AVj in both groups, as previously reported^4,5^. This indicates that the AVj region holds a lower oxygen level than other parts of the heart, strengthening the theory of hypoxic stem cell niche in adult human heart. A hypoxia-responsive element has been identified in the early cardiac transcription factor WT1 sequence that bounds to Hif1α which was required for activation of the WT1 promotor^29^. WT1 has been correlated to epicardial regeneration^30^ as well as expression by endothelial cells^31^. We have previously reported that WT1 is expressed in the human AVj but not in LV cardiomyocytes^4^. In the present study, the co-expression of WT1 was found in small SSEA4+/cTnT+ myocytes in AVj. The numbers of WT1+/cTnT+ cells increased in the AVj after cardiac arrest, interpreted as a regenerative response to global hypoxia in the niche region. Another observation was increased numbers of WT1+/cTnT− cells in LV after cardiac arrest (data not shown) interpreted as an activation of non-myocytes.

It is common for cardiomyocytes to have more than one nucleus. The nuclei are separated from each other in cardiomyocytes. The analyses of PCM1 expression revealed twin nuclei in cardiomyocytes. A systematic quantification of multiple large images showed that the number of twin nuclei increased after cardiac arrest, in both locations. The highest numbers were counted in the LV. Donor 21 was an outlier showing highest number of twin nuclei after the longest period of hypoxia (75 min) compared to the others (Suppl. Table 1). However, it is difficult to draw conclusions from only one case. Binucleation takes place during the fetal development^32^. The absence of Ki67 or PCNA expression in the twin nuclei in the LV suggest that the results represent binucleation rather that proliferation. However, it should be noted that the half-lives of these two proliferation markers are short (~ 1 and 8 h respectively)^20,21^. Although we cannot solidly determine whether the twin nuclei represent ongoing cell division or binucleation, it is worth noting that both these processes reflect mitosis^33,34^. Furthermore, it has been shown that PCM1 is a centrosome protein which localizes to the nuclear membrane^2^ and more specifically to dense structures on the cytoplasmic site of the nuclear envelope^35^. Therefore, the appearance of the PCM1 staining in the twin nuclei with two visible nuclear envelopes (see Fig. 5b2,c2,d) is in itself evidence which strongly suggests binucleation rather than polyploidy within a single nucleus. In line with our results, double nuclei were observed in dedifferentiating cardiomyocytes days after apical resection in newborn mice, whereas neighbouring myocytes which did not undergo dedifferentiation or associated sarcomeric disorganization only displayed single nuclei^36^. Thus, regardless of whether they were destined for cell division or binucleation, the twin nuclei are consistent with a remodeling process.

Neither of the proliferation markers were found in cardiomyocytes in the LV after cardiac arrest, not even in the cardiomyocytes with the low cTnT expression suggesting that remodeling is a longer process, and that proliferation has not been initiated 1–4 days following cardiac arrest. Support for this can be found in the study by Meckert et al. who found 12% of the LV myocytes contained Ki67+ nuclei in 7–13 days-old infarcts. Earlier (1–6 days) and also later (14–21 days), the portion of Ki67+ myocytes was significantly lower^37^. The absence of Ki67+ nuclei in the LV in the present study (1–4 days after cardiac arrest) therefore seems to be largely in agreement with these results.

In contrast to the LV, PCNA and Ki67 were co-expressed with cardiac specific nuclei marker PCM1 in AVj, which may indicate increased proliferation in small myocytes after a period of global hypoxia. Ki67 has a shorter half-time than PCNA^20,21^, which could be an explanation to why more of PCNA+/PCM1+ nuclei compared to Ki67+/PCM1+ nuclei were detected. Another possibility is that PCNA can also be involved in DNA repair, including in human cardiomyocytes^37^. As there were clear examples of PCNA+/PCM1+ as well as Ki67+/PCM1+ twin nuclei in AVj, it appears that at least some of the PCNA positivity was associated with nuclei which had entered the cell cycle. Previously, we reported increased numbers of BrdU+ proliferating cells in the AVj using physical exercise in the adult rats^3^. In addition we have shown expression of biomarkers related to hypoxia, cardiac stem cells, proliferation and migration in the left and right AVj^4,5^ indicating that this region is of importance to cardiomyocyte cell renewal in human. I the current study, the increased expression of proliferation markers in the AVj after cardiac arrest suggests that more cardiomyocytes might had entered the cell cycle.

What may be the ultimate fates of the PCM1+ cardiomyocytes in AVj that displayed cell cycle markers? Regarding some of the PCM1+ nuclei that displayed no clear PCM1+ nuclear envelopes (Fig. 4a2,a3), these are admittedly difficult to interpret. However, there is evidence to suggest that the insoluble perinuclear matrix remains in most phases of the cell cycle but disassembles only in pro-metaphase and metaphase of mitosis, making it possible to visualize myocyte nuclei almost throughout the whole cell cycle^38^. It thus seems possible that some of the Ki67+/PCM1+ and PCNA+/PCM1+ nuclei in the AVj in the present study were in prometaphase and metaphase.

In a study on infarcted human hearts, a low number of Ki67+ myocytes in the periinfarct zone had appearances consistent with conventional mitosis^37^. Thus, there is a slight possibility that minor portion of the Ki67+/PCM1+ and PCNA+/PCM1+ nuclei in the AVj may represent conventional cell division. However, Meckert et al. reported evidence to suggest that in human infarcts, entrance of cardiomyocytes into the cell cycle is transient and that endomitosis, leading to polyploidy rather than mitosis, is the final fate of cycling cells^37^. Nevertheless, since cardiac arrest and myocardial infarction are different conditions, there is a clear need for further studies into these issues. A possible explanation behind the differences between the AVj and the LV in the present study may be that the cardiomyocytes in the AVj are younger and in a more immature stage and thus perhaps able to express proliferation markers early after global ischemia.

Some limitations of the present study should be acknowledged. Immunohistochemistry data shows only a snapshot in time but provide important insights on co-expression of biomarkers in human adult cardiomyocytes. The low number of individuals and the limited range of the reperfusion period after cardiac arrest, as well as the short half-life of the chosen proliferation markers, makes it challenging to ascertain whether the twin nuclei were destined for binucleation, polyploidization or cell division. Also, some of the Ki67 and PCNA positivity may have been reflective of polyploidization and/or DNA damage, both of which may have occurred to varying extents. The methods and markers that we used did not allow us to investigate whether this was indeed the case. The physiological significance of the increased number of twin-nuclei as well as the Ki67+/PCM1+ and PCNA+/PCM1+ nuclei in and the remodelling cardiomyocytes after cardiac arrest thus needs further investigation. Nevertheless, the material is highly unique and may provide important insights into cellular response to cardiac arrest in human heart and clues for therapies aimed at improving heart regeneration.

## Conclusion and clinical significance

Human left ventricular cardiomyocytes do not express stem cell biomarkers under normal conditions. After global hypoxia caused by cardiac arrest, expression of different stem cell biomarkers was found in a subpopulation of left ventricular cardiomyocytes. Notably, the same cells also showed a low expression of cTnT. In addition, we observed an activation of the stem cell niche region with higher expression of stem cell- and proliferation associated markers. Our data thus suggest that cardiomyocytes undergo a regeneration-associated remodelling in response to hypoxia. Dedifferentiation during myocardial repair might provide cardiomyocytes with additional plasticity, enabling survival under hypoxic conditions and increasing the propensity to enter the cell cycle. With additional research, this insight could potentially be a way to increase cardiac stem cell activation for repair after cardiac injury. Finally, since no histological changes were observed, this provides additional evidence that donor hearts with cardiac arrest can be safely used for transplantation.

### Supplementary Information

Supplementary Figures.

Supplementary Table 1.

## Data Availability

The datasets used and analysed during the current study is available from the corresponding author on reasonable request.
