# Supplementary material for: Global ischemia induces stemness and dedifferentiation in human adult cardiomyocytes after cardiac arrest
Source: Sci Rep. 2024 Jun 20;14:14256. doi: 10.1038/s41598-024-65212-z (PMC11190235; doi:10.1038/s41598-024-65212-z)
Supplement: Supplementary file 1 — Supplementary Figures. [file 41598_2024_65212_MOESM1_ESM.pptx]

## Slide 1
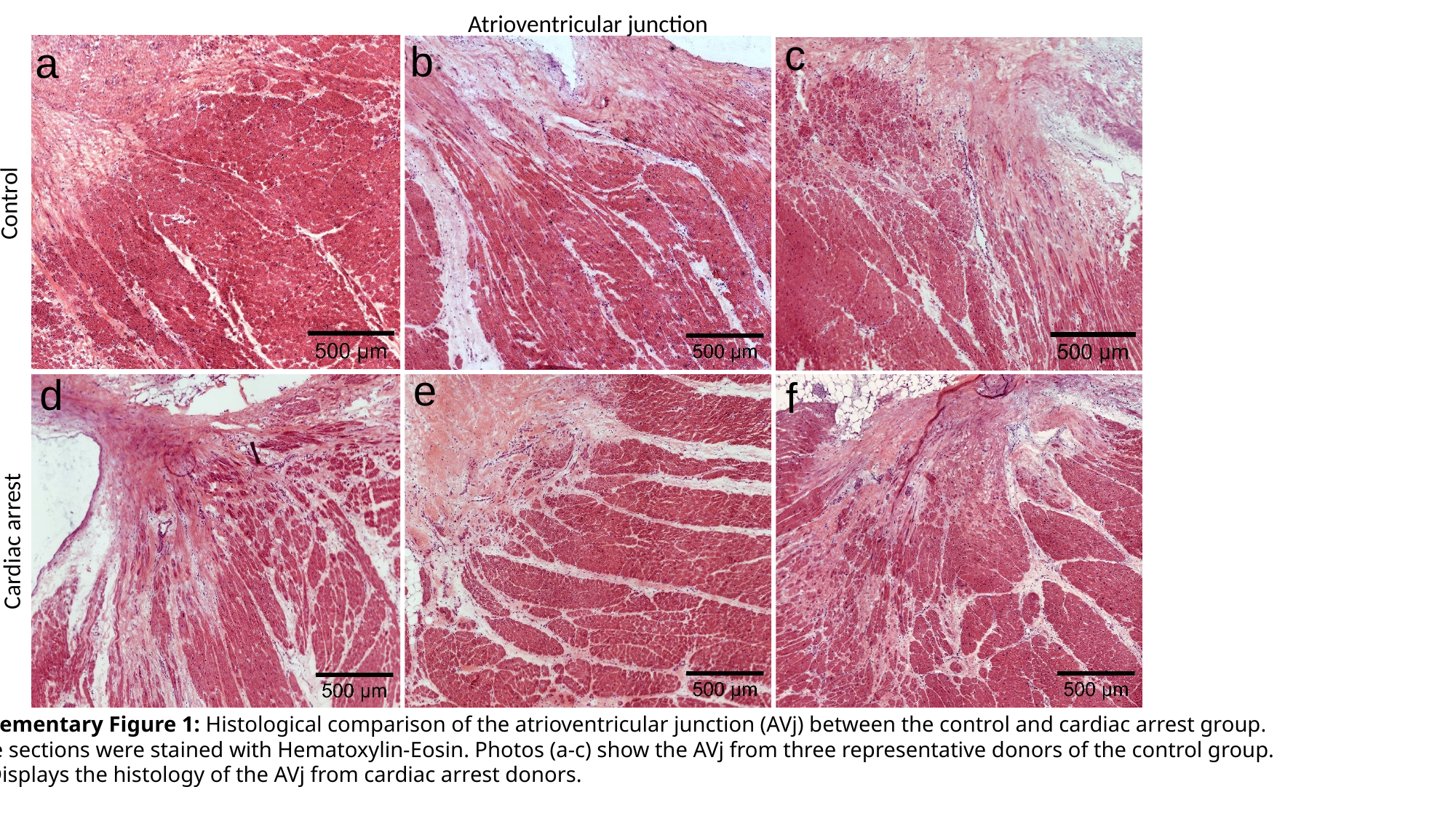

Atrioventricular junction
a
c
b
a
Control
e
d
f
Cardiac arrest
Supplementary Figure 1: Histological comparison of the atrioventricular junction (AVj) between the control and cardiac arrest group.
Tissue sections were stained with Hematoxylin-Eosin. Photos (a-c) show the AVj from three representative donors of the control group.
(d-f) Displays the histology of the AVj from cardiac arrest donors.

## Slide 2
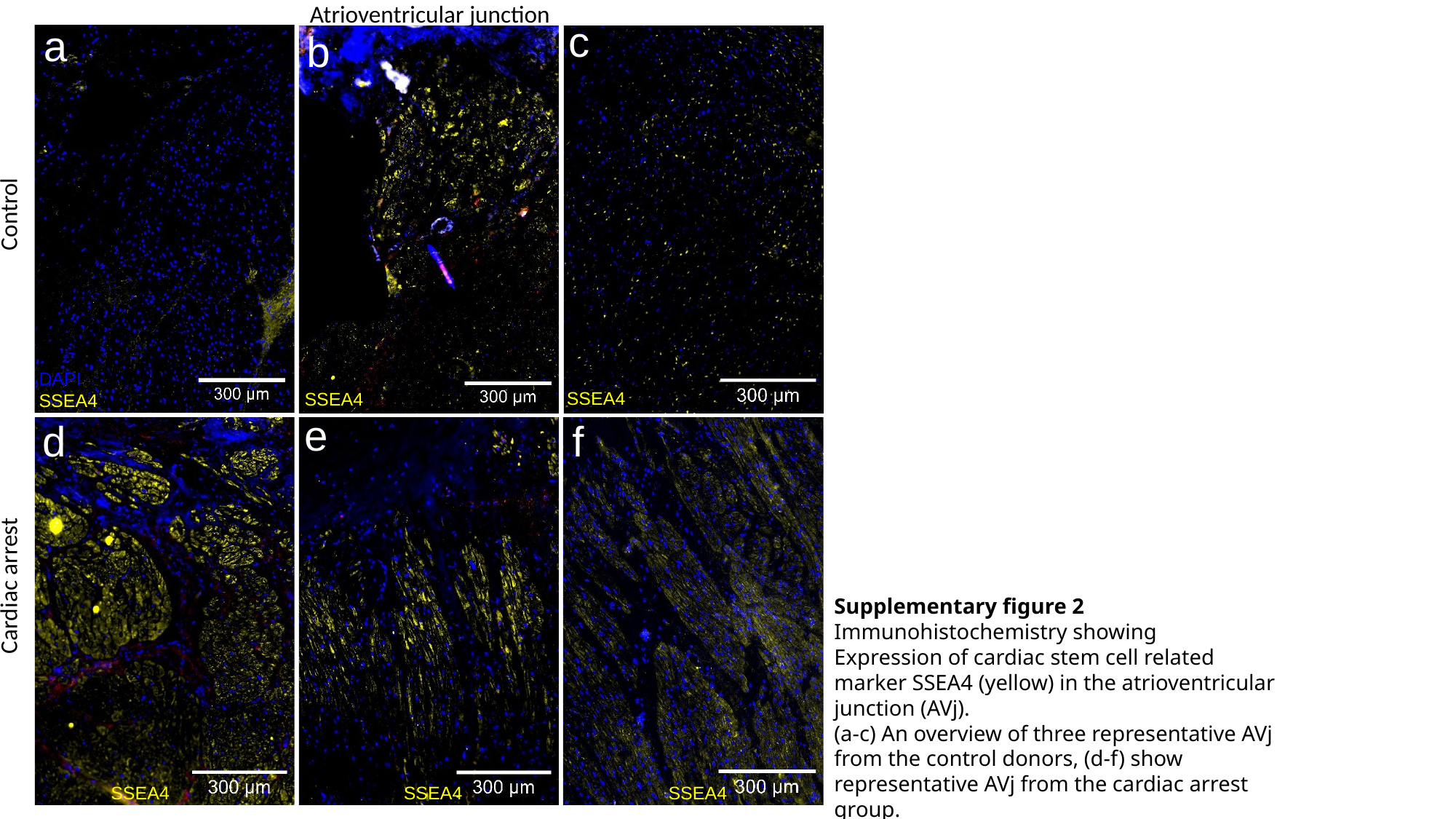

Atrioventricular junction
c
a
b
Control
DAPI
SSEA4
SSEA4
SSEA4
e
f
d
Cardiac arrest
Supplementary figure 2
Immunohistochemistry showing
Expression of cardiac stem cell related marker SSEA4 (yellow) in the atrioventricular junction (AVj).
(a-c) An overview of three representative AVj from the control donors, (d-f) show representative AVj from the cardiac arrest group.
Nuclei were stained blue with DAPI.
SSEA4
SSEA4
SSEA4
